# Imaging appropriateness in pediatric emergency department using the American College of Radiology criteria: an observational study

**DOI:** 10.1007/s10140-026-02452-8

**Published:** 2026-05-12

**Authors:** Michele Carella, Marcello Benevento, Davide Ferorelli, Giandomenico Stellacci, Antonello Sacco, Alessandro Dell’Erba, Biagio Solarino

**Affiliations:** 1 Section of Cardiology, University of Foggia, Foggia, Italy; 2 Department of Legal Medicine and Risk Management, Regional Health Authority of Matera, via Montescaglioso, Matera, 75100 Italy; 3 Section of Legal Medicine, University of Bari, Bari, Italy; 4 Radiology Department, “Giovanni XXIII” Pediatric Hospital of Bari, Bari, Italy

**Keywords:** Pediatrics, Radiology, Diagnostic appropriateness

## Abstract

**Background:**

Even though children are typically more susceptible to radiation-induced illnesses than adults, pediatric radiology is an essential part of contemporary practice. Consequently, one of the most important performance metrics for patient safety is the appropriateness of radiologic procedures. The appropriateness criteria of the American College of Radiology (ACR) are evidence-based guidelines designed to assist referring physicians and other healthcare providers in decision-making regarding diagnostic imaging.

**Methods:**

Using ACR criteria as a reference, this study attempts to assess the suitability of radiologic procedures sought by pediatric emergency physicians. Furthermore, the investigation ought to pinpoint and emphasize possible causes of improper requests. A trainee operator consecutively collected 462 requests for radio diagnostic imaging for neurological diseases of the emergency department of an Italian pediatric hospital and used the pediatric panel of ACR criteria to rate the appropriateness of each request. RESULTS. Due to the absence of crucial clinical information, 24.7% of the requests were not complete. Of the complete requests, only 16.1% were classified as “usually appropriate”, 29.9% as “may be appropriate”, and 54.0% as “usually not appropriate”. CT requests were commonly inappropriate (55.7%, p < 0.01).

**Conclusion:**

Overuse of CT scans can result in costly procedures and unwarranted radiation exposure. In pediatric practice, communication between radiologists and emergency physicians should encourage the use of evidence-based decision-making.

## Introduction

Medical radiologic procedures represent the primary cause of radiation exposure for children [1]. Radio diagnostic examinations are increasingly employed in pediatric emergency departments, allowing for the rapid diagnosis of potentially life-threatening pathologies [2].

Despite this, children are more liable than adults to radiation-induced cancer, due to their higher susceptibility to radiation and their longer expected lifespan post-radiation exposure [3]. Several studies highlighted the risk of radio-induced malignancy in pediatrics [4], [5]. Therefore, appropriateness of diagnostic imaging represents a key performance indicator for patient safety [6]. Nevertheless, many authors showed a widespread overuse of radiological examination in clinical practice, reporting that almost 25% of CT scans performed in the general population are inappropriate [7] [8] (Bouëtté et al. [9]). 

Efforts have been made to enhance the quality and appropriateness of radiologic procedures. In 1993, the American College of Radiology (ACR) developed the appropriateness criteria, which have led, when correctly applied, to an improvement of the quality of procedure requests, minimized exposure to ionizing radiation, and reduced costs [10]. The ACR appropriateness criteria are evidence-based guidelines designed to assist referring physicians and other healthcare providers in decision-making regarding diagnostic imaging. These guidelines are developed and reviewed annually by expert panels specializing in diagnostic imaging and interventional radiology. As of 2023, the ACR criteria covers 233 Diagnostic Imaging and Interventional Radiology topics, encompassing over 1100 variants. Additionally, within the Diagnostic Imaging domain, these criteria address 3000 distinct clinical scenarios.

This study aims to evaluate the appropriateness of radio diagnostic imaging requested by pediatric emergency physicians using ACR criteria as a benchmark. Additionally, the analysis should identify and highlight potential factors contributing to inappropriate requests.

## Materials and methods

### Design and population

The analysis was conducted at a pediatric hospital (250 beds). The authors collected the requests for radio diagnostic imaging for neurological diseases in the emergency department.

The analysis consecutively included 462 pediatric patients presenting one of the following neurological diseases: ataxia, back pain, cerebrovascular diseases, head trauma, headache, and seizure.

### Procedure and outcomes

The ACR appropriateness criteria are based on different clinical scenarios. A rating table provides the appropriateness category (Usually Not Appropriate, May Be Appropriate, Usually Appropriate) and relative radiation level of the different imaging procedures for each clinical scenario.

A trainee operator (graduating medical student in a 6-year medical curriculum) consecutively collected all the requests for radio imaging coming from the emergency department from 20 Jul 2022 to 19 Nov 2023. The operator used the rating tables of the pediatric panel of ACR criteria related to the selected neurological signs/symptoms (Ataxia-Child, Back Pain-Child, Cerebrovascular Disease-Child, Head Trauma-Child, Headache-Child, Seizures-Child, Suspected Spine Trauma-Child) to rate the appropriateness of each request. Expert radiologist consultation helped resolve ambiguous cases.

The imaging request was labeled as “incomplete” when the operator could not choose a rating table because the clinical data in the request were incomplete or ambiguous.

For each diagnostic imaging request, authors recorded the following data: patient’s age, date and time of the request, work shift, applicant’s stated urgency level (Non urgency; Deferrable urgency; Urgency; Emergency), and clinic notes.

Repeated exams on the same patient during the study period were excluded. Only the first imaging exam was included for each patient.

### Statistics

The statistical analysis was performed using Jamovi software 2.3.28. Quantitative variables are expressed as mean, standard deviation, and interquartile ranges; the normality of distribution was verified through the joint interpretation of Q-Q plots, skewness, and kurtosis. The distribution of qualitative variables was evaluated with the χ2-test. The local institutional review board approved this study.

## Results

The study included 462 imaging requests for the same number of patients (248 males, 214 females). Patients’ age ranged between 0 and 17 years old (mean ± SD: 8.75 ± 5.20). (Table 1) No cases were included in the “Suspected Spine Trauma-Child” category.

**Table 1:** Patient and procedure characteristics

Clinical presentation	Mean age	Gender (%)		Triage assessment (%)				Requested procedure (%)		
		M	F	White	Green	Yellow	Red	CT	MRI	X-ray
Ataxia	11.7	1 (33.3)	2 (66.7)	1 (33.3)	2 (66.7)	0 (0.0)	0 (0.0)	3 (100.0)	0 (0.0)	0 (0.0)
Back Pain	12.2	7 (41.2)	10 (58.8)	4 (23.5)	12 (70.6)	1 (5.9)	0 (0.0)	7 (41.2)	2 (11.8)	8 (47.1)
Headache	11.1	61 (48.8)	64 (51.2)	9 (7.2)	94 (75.2)	21 (16.8)	1 (0.8)	124 (99.2)	1 (0.8)	0 (0.0)
Seizures	7.03	50 (56.2)	39 (43.8)	4 (4.5)	62 (69.7)	22 (24.7)	1 (1.1)	88 (98.9)	1 (1.1)	0 (0.0)
Cerebrovascular disease	10.6	11 (40.7)	16 (59.3)	2 (7.4)	14 (51.9)	11 (40.7)	0 (0.0)	27 (100.0)	0 (0.0)	0 (0.0)
Head Trauma	7.45	118 (58.7)	83 (41.3)	18 (9.0)	134 (66.7)	49 (24.4)	0 (0.0)	200 (99.5)	1 (0.5)	0 (0.0)

Table 2 shows how the authors labeled each request according to the ACR guidelines variants (Table 2).

**Table 2:** Clinical variants according to ACR guidelines

Clinical presentation	Variant	Count (%)
Ataxia – child	1 – Child. Acute ataxia, no history of recent trauma. Initial imaging.	3 (0.9)
Back Pain – child	1 – Child. Back pain with none of the following clinical red flags: constant pain, night pain, radicular pain, pain lasting > 4 weeks, abnormal neurologic examination. Initial imaging evaluation.	1 (0.3)
	2 – Child. Back pain with 1 or more of the following clinical red flags: constant pain, night pain, radicular pain, pain lasting > 4 weeks, abnormal neurologic examination. Initial imaging evaluation.	6 (1.7)
	4 – Child. Back pain with 1 or more of the following clinical red flags: constant pain, night pain, radicular pain, pain lasting > 4 weeks, abnormal neurologic examination. Positive radiographs.	6 (1.7)
	6 – Child. Back pain associated with suspected inflammation, infection, or malignancy.	1 (0.3)
Headache – child	1 – Child. Primary headache. Initial imaging.	25 (7.2)
	2 – Child. Secondary headache. Initial imaging.	33 (9.5)
	3 – Child. Sudden severe headache (thunderclap headache). Initial imaging.	1 (0.3)
	4 – Child. Headache attributed to infection. Initial imaging.	11 (3.2)
	5 – Child. Headache attributed to remote trauma. Initial imaging.	1 (0.3)
Seizures	2 – Children 6 months to 5 years of age. Simple febrile seizures. Initial imaging.	9 (2.6)
	3 – Children 6 months to 5 years of age. Complex febrile seizures. Initial imaging.	9 (2.6)
	4 – Children 1 month to 17 years of age. Post-traumatic seizures, not including abusive head trauma. Initial imaging.	1 (0.3)
	5 – Children 1 month to 17 years of age. Focal seizures, not including abusive head trauma. Initial imaging.	4 (1.1)
	6 – Children 1 month to 17 years of age. Primary generalized seizure (neurologically normal). Initial imaging.	53 (15.2)
	7 – Children 1 month to 17 years of age. Generalized seizure (neurologically abnormal). Initial imaging.	7 (2.0)
	8 – Children 1 month to 17 years of age. Intractable seizures or refractory epilepsy.	2 (0.6)
Cerebrovascular diseases – child	1 – Child age older than 6 months. Emergent imaging for clinical presentation suggestive of acute nonsickle cell related stroke. New focal fixed or worsening neurologic defect lasting less than 24 h from last seen normal state. No contraindications to emergent intervention. Initial imaging.	17 (4.9)
	2 – Child. Clinical presentation is suggestive of acute stroke, not a candidate for emergent intervention. Initial imaging.	3 (0.9)
	3 – Child. Clinical presentation suggestive of acute stroke, known or suspected arteriopathy, or moyamoya. Not a candidate for emergent treatment. Initial imaging.	1 (0.3)
	8 – Child. Clinical presentation suggestive of acute stroke, known or suspected high-flow vascular anomaly. Initial imaging.	3 (0.9)
Head trauma – child	1 – Child. Minor acute blunt head trauma. Very low risk for clinically important brain injury per PECARN criteria. Excluding suspected abusive head trauma. Initial imaging.	21 (6.0)
	2 – Child. Minor acute blunt head trauma. Intermediate risk for clinically important brain injury per PECARN criteria. Excluding suspected abusive head trauma. Initial imaging.	89 (25.6)
	3 – Child. Minor acute blunt head trauma. High risk for clinically important brain injury per PECARN criteria. Excluding suspected abusive head trauma. Initial imaging.	34 (9.8)
	4 – Child. Moderate or severe acute blunt head trauma (GCS less than or equal to 13). Excluding suspected abusive head trauma. Initial imaging.	1 (0.3)
	5 – Child. Subacute blunt head trauma with cognitive or neurologic signs.	6 (1.7)

Due to the lack of clinical information, 114 of the 462 included requests (24.7%) were labeled incomplete, and it was not possible to evaluate them according to ACR guidelines. A significant correlation (*p* < 0.001) was observed between clinical scenarios and sufficiency of clinical information. The information was satisfactory in 100% of ataxia cases, 95.5% of seizure cases, 88.9% of cerebrovascular disease cases, 82.4% of back pain cases, 75.3% of head trauma cases, and only 56.8% in headache cases.

The requests with sufficient clinical data were classified as follows: 188 (54.0%) were labeled as “Usually not appropriate”, 104 (29.9%) as “May be appropriate”, and 56 (16.1%) as “Usually appropriate”. [Table 3]

**Table 3:** Requests’ evaluation according to ACR appropriateness criteria

Clinical presentation	Procedure	Lack of clinical data	Usually not appropriate	May be appropriate	Usually appropriate	TOTAL
Ataxia	CT	0	0	1	2	3
	MRI	0	0	0	0	0
	X-ray	0	0	0	0	0
Back pain – child	CT	2	0	5	0	7
	MRI	0	0	1	1	2
	X-ray	1	1	0	6	8
Headache – child	CT	54	41	27	2	124
	MRI	0	0	0	1	1
	X-ray	0	0	0	0	0
Seizures	CT	4	74	9	1	88
	MRI	0	0	1	0	1
	X-ray	0	0	0	0	0
Cerebrovascular diseases – child	CT	3	9	2	13	27
	MRI	0	0	0	0	0
	X-ray	0	0	0	0	0
Head trauma – child	CT	50	63	58	29	200
	MRI	0	0	0	1	1
	X-ray	0	0	0	0	0
TOTAL (%)		114 (24.7)	188 (54.0)	104 (29.9)	56 (16.1)	462

As shown in Tables 3 and 187 (53.9%) of CT scan requests were labeled as “Usually not appropriate” (*p* < 0.001). According to the clinical presentation, the requests were labeled as “Usually not appropriate” in 87.1% of seizure cases, 57.7% of headache cases, 41.7% of head trauma cases, 37.5% of cerebrovascular disease cases, and 7.1% of back pain cases (*p* < 0.001).

## Discussion

Diagnostic radiology plays an essential role in modern medical practice, providing clinicians with critical tools for the effective management of patients. The selection of the appropriate procedure is inherently complex, requiring physicians to carefully consider the cost, diagnostic efficacy, and potential risks associated with each procedure. On the other hand, the inappropriate use of diagnostic imaging can result in unnecessary radiation exposure for patients.

Even though radiologists have the most thorough knowledge of the advantages and disadvantages of different radiological procedures, they sometimes have little direct control over the choice of imaging studies [11]. Moreover, it is often non-radiologists who prescribe radiologic s [12]. 

The results showed that 24.7% of requests lack any essential clinical information, which could lead to inappropriate utilization of imaging resources, lower accuracy, and misdiagnoses [13]. The backbone of any diagnostic request should include three key points: 1) a clinical question, 2) localization of signs and symptoms when appropriate, and 3) relevant clinical history [14]. All the lacking requests provided an insufficient clinical history. Many requests simply outline the clinical case with the statement “examination requested by neurology specialist” or similar. As a consequence, clinical details, such as duration, intensity, the presence or absence of accompanying symptoms, signs, and diagnostic suspicions, are often omitted.

The lack of clinical information was common in cases of headache (43.2%). Given that the CT indication for headaches relies on symptoms, the absence of clinical information may result in inappropriate examinations. These findings are consistent with existing literature, wherein authors have correlated the adequacy of clinical information in requests with radiological appropriateness [15, 16]. On the other hand, we found sufficient clinical information in 95,5% of seizures, 88,9% of cerebrovascular diseases, and 75,1% of head trauma cases.

Among those requests that included sufficient clinical information, CT scan requests were inappropriate more often than other procedures, as 53.9% of requests were inappropriate. This is in line with the literature, as showed that 20–54% of CT requests were inappropriate in pediatrics [17]. 

The high percentage of inappropriate exams may be attributable to defensive medicine practice, which represents a serious and common challenge all over the world [18]. Although difficult to measure precisely, it is widely believed that this practice accounts for a quarter of total imaging expenses [19]. While this study did not specifically aim to assess the reasons for the overuse of imaging, it is important to recognize that such overuse is often linked to fear of litigation, particularly in emergency department settings [20, 21]. 

Other possible reasons for the overuse of CT scans may result from physicians’ belief that radiologic s are essential for diagnosis [22]. Moreover, patient or family pressure, limited access to alternative diagnostic methods, such as MRI, ultrasound or lack of trained personnel to perform alternative diagnostic tests, and limited time available per patient in an overloaded emergency system may contribute to CT scan overuse [23, 24] [25, 26]. 

Our study showed excessive use of CT scans, especially in cases of headaches or epilepsy. So, physicians tend to overprescribe CT scans despite guidelines from the ACR indicating their limited utility compared to observation or MRI.

In cases of head trauma, back pain, and cerebrovascular disease, prescribed exams are more frequently appropriate. The request’s appropriateness is higher when operators prescribe MRI exams compared to CT scans.

Inappropriate exams lead to overexposure to radiation, and this represents a significant risk, particularly for growing patients who are more susceptible to the long-term stochastic effects of radiation. Prescription of more appropriate exams, such as MRI or clinical observation, would have significantly reduced radiant overexposure and respected the ALARA principle.

The ALARA principle, which stands for “as low as reasonably achievable”, is a cornerstone in radiology and invites minimizing radiation exposure while ensuring high-quality diagnostic images for clinical purposes [27]. These fundamental interventions will substantially enhance children’s safety by employing ALARA strategies such as following guidelines like ACR, considering alternative non-ionizing tests, and using low-dose scan protocols [28]. 

suggest that radiologists should exercise greater supervision over examination requests and reject any inappropriate ones [29]. However, our viewpoint finds this strategy unproductive and unfeasible. Given their busy schedules and limited access to full patient information, radiologists may not be able to effectively carry out this responsibility.

One potential solution, in our view, is to improve communication between emergency physicians and radiologists, possibly using diagrams and protocols based on the latest evidence. Such measures could enhance not only the quality of clinical information but also prescription appropriateness.

The gathered cases were assessed by a single operator in accordance with ACR standards. While ensuring good uniformity in assessment, this could lead to a great deal of subjectivity. This limitation should be taken into account in future research. The authors examined requests for radiological exams to analyze the transfer of information between physicians in different departments. No data were collected on the exams actually performed following the request.

## Conclusions

The study highlighted the excessive and often inappropriate use of computed tomography (CT) for neurologic diseases in a pediatric emergency department, leading to an increased risk of radiation overexposure in children.

Furthermore, the study revealed a lack of attention to the clinical information accompanying imaging requests, which is frequently incomplete, particularly in less urgent cases. This deficiency may compromises communication quality and hinders the radiologist’s ability to suggest alternative diagnostic options.

In our opinion, it is essential to prioritize the development of educational initiatives that emphasize evidence-based decision-making and raise awareness of ACR guidelines, which may help reduce the overutilization of CT scans. Lastly, promoting effective communication between emergency physicians and radiologists can support the exchange of valuable clinical information, leading to more precise and suitable imaging requests, improved patient care, cost-effectiveness, and minimized long-term health risks, especially in the vulnerable pediatric population.

## Data Availability

Data available on request from the authors.
